# A firm recommendation: measuring the softness of infant sleep surfaces

**DOI:** 10.1186/s40621-021-00325-x

**Published:** 2021-09-13

**Authors:** Sheena H. Gillani, Gina S. Lowell, Kyran P. Quinlan

**Affiliations:** 1 Chicago Medical School, Rosalind Franklin University of Medicine and Science, 3333 Green Bay Rd, North Chicago, IL 60064 USA; 2 Department of Pediatrics, Rush University Children’s Hospital, 1653 W. Congress Pkwy, Chicago, IL 60612 USA

**Keywords:** SUID, Soft bedding, Safe sleep, Mattress softness, Mattress firmness

## Abstract

**Background:**

Approximately 3600 sudden unexpected infant deaths (SUID) occur annually in the United States, and a quarter of SUIDs are caused by unintentional suffocation and strangulation in bed, with soft bedding use being a significant risk factor. Therefore, The American Academy of Pediatrics (AAP) recommends infants sleep on a “firm” surface, though neither an objective definition nor national standard has been established. The purpose of this study is to report on the performance of a device that measures mattress softness and to provide quantitative values of softness for various infant sleep surfaces.

**Methods:**

In collaboration with the authors and a national child product safety organization (Kids in Danger), University of Michigan engineering students designed and validated a device that measures the vertical depression (softness) of a simulated 2-month-old’s head on a sleep surface. A total of 17 infant sleep surfaces − 14 household surfaces and 3 hospital mattresses - were measured between April 2019 and January 2020. The average softness of each surface was calculated. Surfaces were also measured with soft bedding, which included an infant fleece blanket, and firm and soft pillows.

**Results:**

The average softness for the 14 household sleep surfaces ranged from 7.4–36.9 mm. The 2019 cribette playard and the 2018 infant spring had similar softness (21 mm) as the 2018 and 2019 adult foam and 2015 sofa. An infant’s fleece blanket folded once added an additional 2.3–6.5 mm of softness, folded twice added 4.8–11.6 mm, and folded three times added 11–21.8 mm. Using a firm pillow added 4.0–20.9 mm of softness while using a soft pillow added 24.5–46.4 mm. The softness for the 3 hospital sleep surfaces ranged from 14 to 36.9 mm, with the infant bassinet being the firmest and the pediatrics mattress being the softest.

**Conclusions:**

We found a wide range of softness among sleep surfaces, with some infant mattresses as soft as some adult mattresses. Adding blankets and pillows to mattresses measurably increased softness. Quantifying sleep surface softness will advance our understanding of how softness relates to SUID risk. We hope this new information will further inform safe infant sleep recommendations and improve mattress safety standards nationally.

**Supplementary Information:**

The online version contains supplementary material available at 10.1186/s40621-021-00325-x.

## Background

During the 1990s, the “Back to Sleep” campaign helped reduce deaths attributed to sudden infant death syndrome (SIDS); however, progress plateaued by 1999. Since then, approximately 3600 sudden unexpected infant deaths (SUIDs) have occurred annually in the United States (Sudden Unexpected Infant Death and Sudden Infant Death Syndrome 2020). A quarter of SUIDs are caused by unintentional suffocation and strangulation in bed, with soft mattresses and soft bedding use being significant risk factors (Sudden Unexpected Infant Death and Sudden Infant Death Syndrome 2020; Mitchell et al. 1996; Hauck et al. 2003; SIDS and Other Sleep-Related Infant Deaths; *Pediatrics.*; Kemp et al. 1998; Scheers et al. 1998; Carpenter and Shaddick 1965; Adler et al. 2006; Brooke et al. 1997; Kemp et al. 1994). Therefore, The American Academy of Pediatrics (AAP) and the US Consumer Product Safety Commission (CPSC) recommend infants sleep on a “firm” mattress, with a fitted sheet, and no soft objects in the sleeping area (*Pediatrics.*; CPSC 2012; CPSC Safety Alert 2013). The AAP further defines a firm surface to be one that retains its shape and does not conform to an infant’s head while a soft surface becomes indented by an infant’s head (*Pediatrics.*). Additionally, the AAP clarifies “soft bedding” to include pillows, quilts, comforters, loose bedding, and soft toys (*Pediatrics.*; Kemp et al. 1998; Kemp et al. 2000). However, neither the AAP or the CPSC provide an objective definition or means to measure firmness, and no national standard of mattress “firmness” has been established.

Mattress firmness is just one piece of the complicated SUIDs puzzle. Death scene investigation studies typically find infants sleeping on soft sleep surfaces, in the prone position, and/or with soft bedding (Mitchell et al. 1996; Hauck et al. 2003; Kemp et al. 1998; Scheers et al. 1998; Carpenter and Shaddick 1965; Brooke et al. 1997; Kemp et al. 1994; Kemp et al. 2000; Blair et al. 1999). Although having a combination of these can increase SUID risk, a soft mattress is especially dangerous because it conforms to the shape of an infant’s head, creating pockets of space that can cover the infant’s nose and mouth, increasing the risk of suffocation and rebreathing of expired gases (*Pediatrics.*; Kemp et al. 1998; Scheers et al. 1998; Kemp et al. 1994; Kemp et al. 2000; Emery and Thornton 1968). One study of SIDS cases in New Zealand also found that soft infant mattresses had a significantly increased risk of SIDS (OR 2.36; 95%CI 1.06–5.25) compared to firmer mattresses (Mitchell et al. 1996). Many parents are aware of the importance of placing an infant on a firm mattress; however, parents interpret “firmness” differently. For example, a qualitative study of 83 mothers found that some mothers thought they still had a firm mattress even after laying pillows and blankets on top of the mattress because these soft objects were under the fitted sheet (Ajao et al. 2011). For their infant’s comfort, parents often put blankets on the crib or bassinet mattress and place the baby to sleep on top of them. Intuitively this may seem cozy, but it appears to increase SUID risk. Further, it is common for infants to sleep on surfaces other than their own mattresses. These surfaces, such as sofas and adult beds, not only tend to be softer, but also increase the risk of infant suffocation, strangulation, entrapment, and unintentional injury, thus increasing the SUID risk significantly (Hauck et al. 2003; *Pediatrics.*; Adler et al. 2006; Kemp et al. 2000; Blair et al. 1999; Vennemann et al. 2012).

Little has been published on efforts to quantify the softness of infant sleep surfaces. We found few such reports of investigators who developed a manner to quantify the softness of mattresses and other sleep surfaces (Kemp et al. 1994; Schlaud et al. 2010; Somers 2012). Two groups determined the softness by manually measuring the distance an object sank on various sleep surfaces (Schlaud et al. 2010; Somers 2012). Another group calculated the area of contact between an infant mannequin head and various sleep surfaces in order to determine softness (Kemp et al. 1994).

The purpose of this study is to report on the performance of a device developed to measure mattress softness and to provide quantitative values of softness for various infant sleep surfaces, including new and used adult and infant mattresses, sofas, and hospital mattresses and bassinets. Using this device, we also measured the additional softness that soft bedding adds to a sleep surface. We hope this new information will further inform safe infant sleep guidance from national organizations such as AAP and CPSC and improve mattress safety standards nationally.

## Methods

**Fig. 1:**
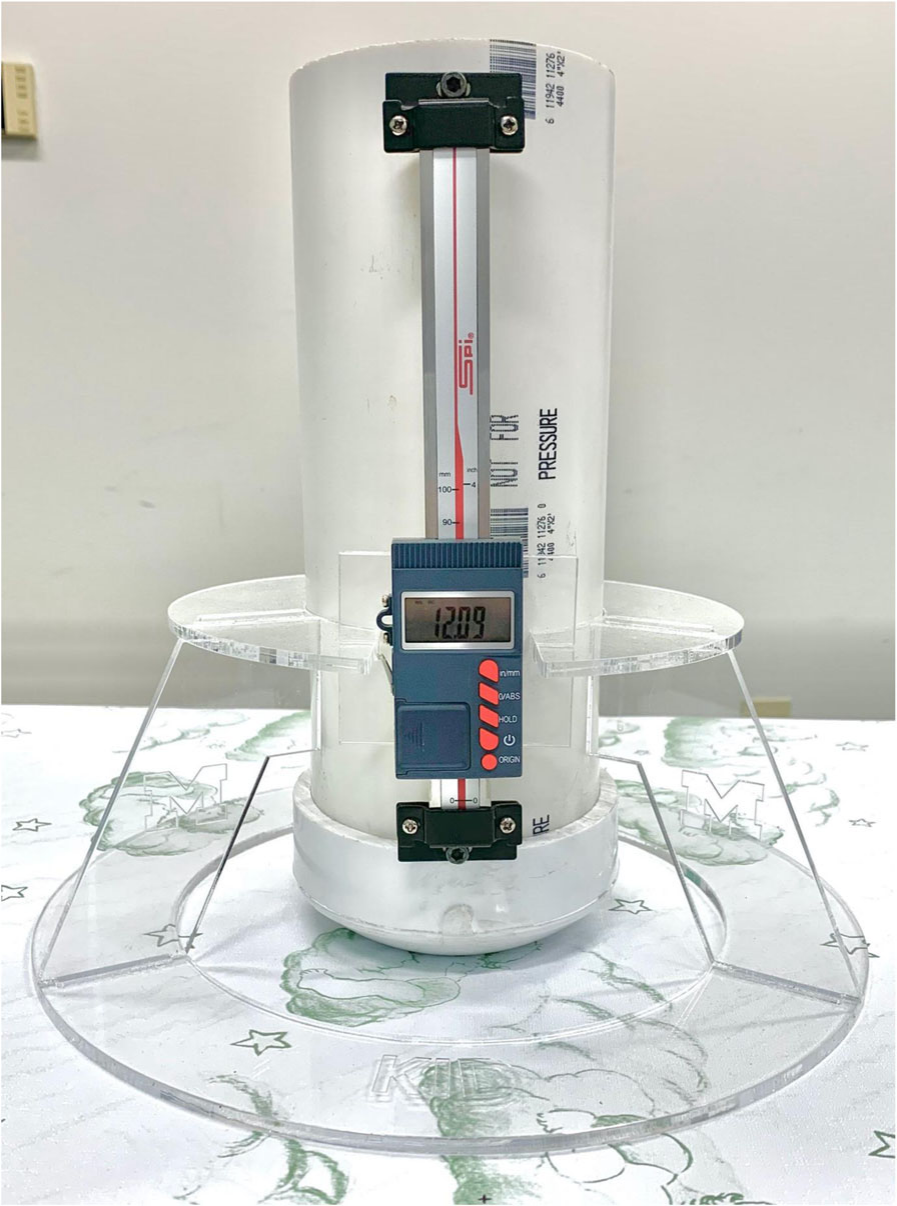
Mattress softness measuring device

In collaboration with the authors and a national child product safety organization (Kids in Danger), University of Michigan engineering students designed and validated a device that simulates the shape and weight of an infant’s head and provides an electronic readout of the vertical indentation that occurs on a surface. The vertical indentation of a 2.5 lb. weight was measured and used to represent the softness of the surface—the higher the measure of vertical indentation, the greater the softness of the surface. A 2.5 lb. weight was used because it was thought to closely resemble the weight of a 2 month’s old head. This particular age was chosen because that is the peak age for SUID risk. Structurally, the diameter of the device’s outer base is 32 cm and the diameter of the inner base is 21.75 cm. Information on the design and reliability of the mattress measuring device can be found in Supplemental Figure 1, and an image of the device is shown in Fig. 1.

A convenience sample of 17 infant sleep surfaces, including 14 household surfaces and 3 hospital mattresses, were measured between April 2019 and January 2020. The 14 household surfaces include: 1 used and 4 new infant crib and bassinet mattresses, 5 used and 1 new adult mattress, and 3 used sofas. Further details about each surface, including the age, brand, used vs new status, filling material, and size can be found in the Table 1.

**Table 1:** Description of sleep surfaces and soft bedding

Type/Year Manufactured	Brand	Used or New	Filling Material	Size
**Adult Mattresses**				
**Adult Foam 2007**	Harlem Furniture Nobility Ther-A-Pedic	Used	Unknown	King (76″ × 80″)
**Adult Foam 2018**	Tempurpedic ProAdapt Medium	Used	Visco Elastic Polyurethane Foam (57%), Polyurethane Foam (43%)	King (76″ × 80″)
**Adult Foam 2019**	Tempurpedic Adapt Medium	New	Visco Elastic Polyurethane Foam (52%), Polyurethane Foam (48%)	King (76″ × 80″)
**Adult Spring 1994**	Sealy Firm Bilt Deluxe	Used	Unknown	Queen (60″ × 80″)
**Adult Spring 2006**	Simmons Beautyrest Classic	Used	Unknown	Queen (60″ × 80″)
**Adult Spring 2017**	Corsicana Bedding	Used	Polyurethane (52%), Other fibers (26%), Blended Fiber Batting (70% Rayon, 30% Polyster)	Queen (60″ × 80″)
**Infant Mattress**				
**Infant Foam 2016**	Moonlight Slumber	Used	Polyurethane Foam (100%)	Crib (28″ × 52″)
**Infant Cribette Playard & Cribbette Bassinett 2019**	Crib for Kids	New	Polyurethane Foam (100%)	38.5″ × 26”
**Infant Spring 2018**	Dream on Me Inc	New	100% Polyester Fiber Padding	Crib (28″ × 52″)
**Infant Spring 2018 small**	Dream on Me Inc	New	100% Polyester Fiber Padding	26 × 38
**Sofas**				
**Sofa 2006**	Southern Motion Inc	Used	Polyurethane (60%) & Polyester Fiber (40%)	NA
**Sofa 2015**	Man Wah Furniture	Used	Polyurethane (51%) & Polyester Fiber (49%)	NA
**Sofa 2016**	Shenzhen Long Sharp Furniture Co, LTD	Used	Polyurethane Foam Pad (91%), Polyester Fiber Batting (9%)	NA
**Hospital Mattresses**				
**Hospital Infant Bassinet**	Standard Textile Sure Chek	Used	55% Cotton, 45% Polyester	25.75″ × 12″ W
**Hospital Peds**	Unknown	Used	Unknown	Unknown
**Hospital Adult**	Unknown	Used	Unknown	Unknown
**Soft Bedding**				
**Soft Pillow**	American Textile Company - The Big One - Kohl’s	Used	100% Polyester Fiber	Queen
**Firm Pillow**	Carpenter Co - JCPenny Home Collection	Used	100% Polyester Fiber	Queen
**Infant Fleece Blanket**	Unknown	Used	100% Polyester Fiber	Crib

In order to measure softness consistently, the mattress measuring device was placed on each of the fourteen household surfaces by the same person (SG). Each surface was measured three times at each of the three identified locations for a total of 9 measurements per sleep surface. For adult mattresses, the three locations that were measured included the upper right quadrant, upper left quadrant, and center of the mattress. These locations were selected because they were the sites measured by the developers of the device and doing so allowed us to capture the minor differences in softness by location on any one mattress. Additionally, anecdotally, these locations are the most likely areas where caregivers place their infants to sleep. Some parents place their infant in the center of the bed, while others are concerned about placing an infant in between two parents. The latter set of parents rather lay their infant on the side of one parent, between the parent and the wall. These three locations could not be measured on infant mattresses and bassinets because they are shorter and narrower than adult mattresses. The only three locations that could be measured on the infant surfaces were the head, center, and foot of the mattress. For each sofa, the three locations included each of the three seating cushions. Each location was then measured for two minutes. After each two-minute measurement, the device was zeroed and then consecutively placed between the three locations to prevent further indentation of the same area. The mean of all nine measurements was calculated to determine the average softness (mm) of each surface.

Using the same protocol for measuring surface softness, the softness of each surface was measured when an infant fleece blanket, which weighed 500 g, was added on top and folded once, twice, and three times. The same protocol was also used when measuring soft and firm pillows; however, it was only measured on the following four surfaces: Adult Spring 2006, Adult Spring 2017, Adult Foam 2006, Adult Foam 2019. Further, although the soft and firm pillow consisted of the same filling material, the firm pillow had objectively less filling and was flatter than the soft pillow. The average of all three locations (mm) was calculated for each soft bedding item. The additional softness added from soft bedding was also determined by calculating the difference between the softness of the surface with soft bedding and the softness of the surface.

The softness of hospital mattresses was tested at a tertiary academic center. Both the Adult Hospital mattress and the Infant Bassinet were from the Mother Baby Unit while the pediatric mattress was from the general pediatric unit. No soft bedding was measured on any of the hospital mattresses. The same protocol for measuring surface softness was used for these mattresses as well.

## Results

**Fig. 2:**
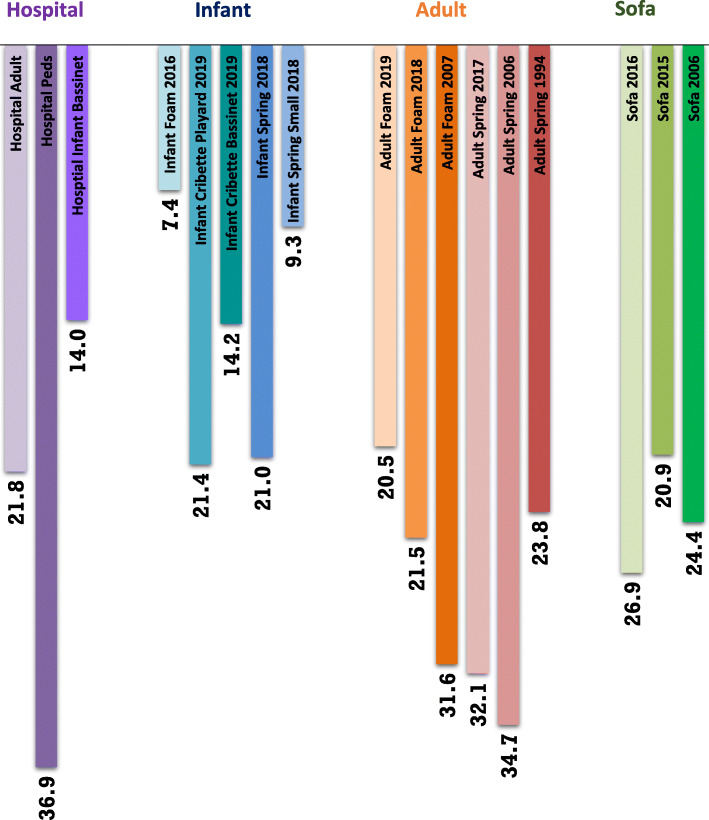
Average softness of sleep surfaces (mm)

Average softness for all 17 sleep surfaces ranged from 7.4 mm (Infant Foam 2016) to 36.9 mm (Hospital Peds). (Fig. 2) The range of softness for infant mattresses was 7.4 mm (Infant Foam 2016) to 21.4 mm (Infant Cribette Playard 2019), for adult mattresses was 20.5 mm (Adult Foam 2019) to 34.7 mm (Adult Spring 2006), and sofas were 20.9 mm (Sofa 2015) to 26.9 mm (Sofa 2016). The 2019 cribette playard and the 2018 infant spring had approximately the same softness (~ 21 mm) as the 2019 adult foam (20.5 mm) and 2015 sofa (20.9 mm). Figure 2 also shows the three hospital mattresses tested. The pediatric mattress was the softest (36.9 mm) of all the surfaces, while the adult hospital mattress (21.8 mm) and the infant hospital bassinet (14.0 mm) had similar softness to the adult and infant home mattresses, respectively.

**Fig. 3:**
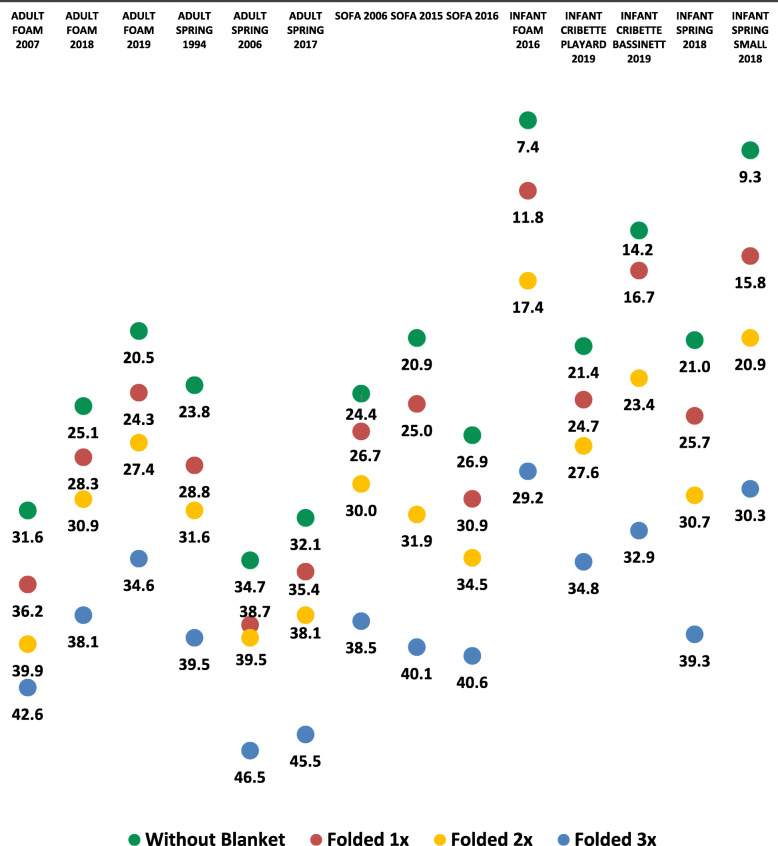
Average softness of sleep surfaces with and without an infant fleece blanket (mm)

The average softness when an infant fleece blanket is folded on top of each of the 14 household sleep surfaces is shown in Fig. 3. When the infant fleece blanket was folded once, the average softness of surfaces ranged from 11.8–38.7 mm, folded twice ranged from 17.4–39.9 mm, and folded three times ranged from 29.2–46.5 mm. When subtracting the average softness of the surface without soft bedding from values in Fig. 3, it was found that an infant’s fleece blanket folded once added an additional 2.3–6.5 mm of softness, folded twice added 4.8–11.6 mm, and folded three times added 11–21.8 mm.

**Fig. 4:**
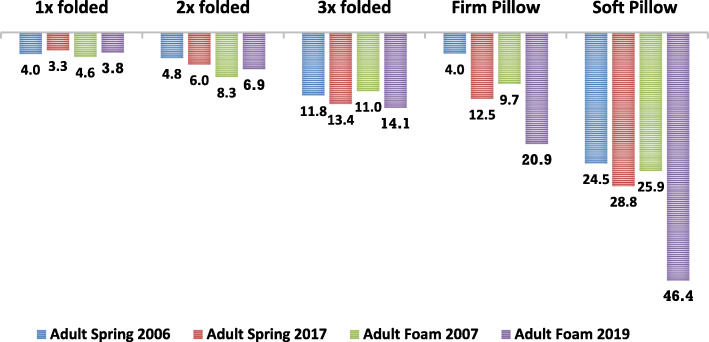
Average added softness of adult mattresses with soft bedding (mm)

The additional softness added by all soft bedding on four specific adult mattresses is shown in Fig. 4. On these four surfaces, folding an infant’s fleece blanket folded once added an additional 3.3–4.6 mm of softness, folded twice added 4.8–8.3 mm, and folded three times added 11.0–14.1 mm. A firm pillow added 4.0–20.9 mm of softness while a soft pillow added 24.5–46.4 mm.

## Discussion

We have described a novel device designed to measure the softness of sleep surfaces. Measuring the softness of a variety of surfaces where infants are often put to sleep showed some surprising results. Some infant mattresses were found to be as soft as some adult mattresses. Some adult mattresses were softer than sofas. The cribette playard was as soft as an adult mattress. Of all the infant and adult sleep surfaces we tested, the softest surface we studied was the pediatric hospital mattress. We demonstrated that adding soft bedding to any sleep surface measurably contributed to sleep surface softness.

Some studies have developed similar initiatives for measuring mattress softness. A German investigation of SIDS death scenes placed a 2 kg weight at the same location on the sleep surface where the infant was found, and then measured how much the weight sank into the mattress (Schlaud et al. 2010). An Australian group tried to replicate the German mattress measuring device using household items that weighed a total of 2.3 kg. Specifically, they placed a dozen CDs tightly bound with cling wrap on top of a mattress surface, and then placed two unopened one-liter milk cartons on top of the CDs (Somers 2012). Another SIDS investigation group in the United States measured mattress softness by calculating the area of contact between an infant mannequin head and the sleep surface (Kemp et al. 1994).

Because Schlaud et. al’s study also used weight to measure mattress firmness, it allows for an easier comparison with our study. Schlaud et al. found that 27 of 41 (67%) scene investigation mattresses had > 14.5 mm indentation. When adjusting for socioeconomic status, nationality, and matching factors, a surface with an indentation of > 14.5 mm was significantly associated with risk of SIDS and had an odds ratio of 4.4 (95% CI 1.1–8.7) (Schlaud et al. 2010). Using a lighter weight for measurements (2.5lbs ~ 1.1 kg), 13 of 17 (76%) surfaces had an indentation > 14.5 mm. All the surfaces that had indentation < 14.5 mm were infant mattresses (Infant Foam 2006, 7.4; Infant Spring Small 2018, 9.3; Hospital Infant Bassinet 14.0; Infant Cribette Bassinet 2019, 14.2). Infant mattresses are expected to be firmer than all other sleep surfaces; however, we found two of our infant sleep surfaces (2018 Infant Spring, 21.0; 2019 Cribette Playard, 21.4) were just as soft as some adult mattresses and sofas.

Further, we found that folding an infant’s fleece blanket on top of the sleep surface measurably increased softness. Folding the blanket once added 2.3–6.5 mm of softness, while folding the blanket twice doubled the softness (4.8–11.6 mm) and folding it three times almost tripled the softness (11–21.8 mm). Schlaud et al. found that 88.5% of death scene cases they studied in Germany were using a duvet (Schlaud et al. 2010). They also found that most cases were using a heavier duvet (> 819.5 g), not a lighter blanket (500 g) found in our study. The use of blankets during infant sleep, however, is common. In a multistate survey conducted in 2010, it was found that 69.3% of infants slept with a light blanket (Ajao et al. 2011). Many mothers are aware of the dangers of suffocation and strangulation with soft bedding use, like blankets; however, some mothers felt that if the blanket was light and not near the head or neck, that it would be safer to use (Ajao et al. 2011).

Little has been previously reported specifically on the softness that pillows add to the infant sleep environment. Our study showed that using a firm pillow added 4.0–20.9 mm of softness while using a soft pillow added 24.5–46.4 mm. This finding contributes to the concern for pillows in the sleep environment posing an especially high risk for SUID. Schlaud et al. found that 41% of SIDS infants were sleeping with pillows compared to only 18% of controls (Schlaud et al. 2010). When controlling for confounders, pillow use was associated with SUID risk with an OR 4.3 (95%CI 1.6–11.6) (Schlaud et al. 2010). Besides comfort, many parents use pillows to prevent their infant from falling, especially if the infant is sleeping in unenclosed areas like adult beds or sofas. Parents may think they are making the sleep environment safer, but using pillows creates a particular hazard (*Pediatrics.*; Scheers et al. 1998; Carpenter and Shaddick 1965; Adler et al. 2006; Kemp et al. 2000; Blair et al. 1999; Emery and Thornton 1968; Ajao et al. 2011; Schlaud et al. 2010). Providing quantitative measures may help parents further understand the risk of adding soft bedding to other sleep surfaces.

We found that sofas and adults mattresses had similar levels of softness, with sofas being firmer than some mattresses. Specifically, we saw that the firmness for adult mattresses ranged from 20.5–34.7 mm while the firmness of sofas ranged from 20.9–26.9 mm. Sleeping on an adult mattress is more common than sleeping on a sofa; however, sofas have been associated with a significantly more increased risk than adult mattresses because of the increased risk of wedging (*Pediatrics.*; Kemp et al. 2000; Blair et al. 1999; Vennemann et al. 2012). One study of SIDS infants in England found that 25.5% of the infants were bed-sharing on an adult mattress while only 6.2% were on the sofa. However, the OR for risk of SIDS controlling for confounding variables was 1.35 (95% CI 0.83–2.20) for bed-sharing and 25.86 (95% CI 6.72–99.47) for sofa sharing (Blair et al. 1999).

Few studies have looked into firmness of hospital mattresses. Kemp et al. found that the conventional bedding, which consisted of a foam crib mattress, a spring crib mattress, and two hospital bassinet mattresses, was significantly firmer than the bedding found at death scenes (Kemp et al. 1994). On the other hand, our study showed the adult mattress (21.8 mm) and the infant bassinet (14.0 mm) were comparably firm to home mattresses. Furthermore, the pediatric hospital mattress was the softest (36.9 mm) of all the surfaces we measured. Ensuring that hospital mattresses are firm is important given hospitalized infants, though typically monitored, are likely at greater risk of SUID given intercurrent illness and the likelihood of other underlying conditions (*Pediatrics.*). Also, studies have shown that parents tend to mimic safe sleep practices that they observed while in the hospital (Walcott et al. 2018; Colson and Joslin 2002). If the infant mattress in the hospital is not as firm as other standard infant mattresses, this may give parents the wrong impression of the correct level of firmness an infant mattress should have.

### Limitations

Limitations of this study are acknowledged. This study measured a limited number and variety of surfaces in our convenience sample. Although the production year for the various sleep surfaces were reported, the use history (i.e., how “used” a surface was) of each surface was unable to be quantified. Also, there is the potential for operator error with placing the instrument onto the surface for each softness measurement. This was reduced by having a single individual (SG) use the device in a consistent manner. Additionally, measuring the softness of smaller products for infant sleep was limited due to the diameter of the device’s outer base (32 cm) as currently designed. Infant products with smaller widths cannot be measured using our device. Lastly, our study was not designed to correlate the relationship between mattress softness and SUID risk. In the future, we plan to use our device to measure sleep surfaces during SUID death scene investigations in order to begin addressing this question. However, other studies have found that softer surfaces had a significantly increased risk of SIDS compared to firmer surfaces (Mitchell et al. 1996; Kemp et al. 1998; Emery and Thornton 1968).

## Conclusion

We have demonstrated that the device we developed provides a reliable quantitative measure of the softness of the surfaces where infants are often put to sleep. We found a wide range of softness among sleep surfaces, with some infant mattresses as soft as some adult mattresses. Adding soft bedding such as folded blankets and pillows to the mattress measurably increased softness. Quantification of infant sleep surface softness makes it possible to confirm infant sleep surfaces follow the recommendation that they are truly “firm.” Future studies include utilizing our mattress measuring device to measure sleep surfaces during SUID death scene investigations, which will allow for a better examination of how the softness of sleep surfaces quantitatively contributes to SUID risk. This work may also aid in our clinical counseling of parents and caregivers how SUID risk relates to soft sleep surfaces.

## Supplementary Information

**Additional file 1: Supplementary Fig. 1.** KID Mattress Measurement Device.

## Data Availability

The datasets used and/or analyzed during the current study are available from the corresponding author on reasonable request.

## Abbreviations

SUID: Sudden unexpected infant death
AAP: The American Academy of Pediatrics
SIDS: Sudden infant death syndrome
CPSC: Consumer Product Safety Commission
KID: Kids in Danger
