# Supplementary material for: A firm recommendation: measuring the softness of infant sleep surfaces
Source: Inj Epidemiol. 2021 Sep 13;8(Suppl 1):30. doi: 10.1186/s40621-021-00325-x (PMC8436463; doi:10.1186/s40621-021-00325-x)
Supplement: Supplementary file 1 — Additional file 1: Supplementary Fig. 1. KID Mattress Measurement Device. [file 40621_2021_325_MOESM1_ESM.doc]

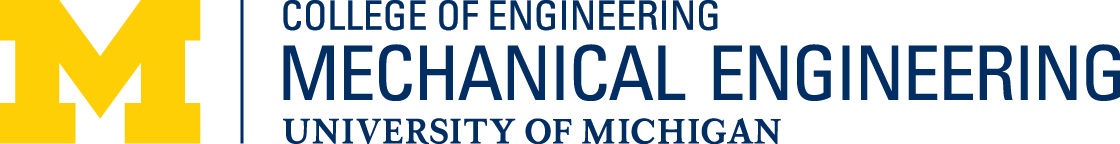


Course Instructor: Amy Hortop (abhortop@umich.edu) Section Instructor: Grant Kruger (ghkruger@med.umich.edu)

# Supplementary Figure 1: KID Mattress Measurement Device

# **ME 450 Team 16**

Regan Ora, Max Nikolai, Conner Levy, Parker Chmiel, Sam Knowles

Contact at: [ME450-KID@umich.edu](mailto:ME450-KID@umich.edu)

**Sponsors: Nancy Cowles, Kids in Danger and Dr. Kyran Quinlan, Rush Hospital**


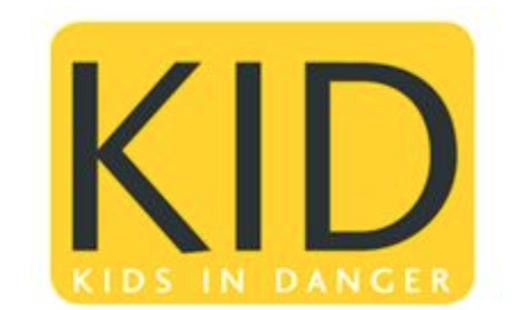
Additional Questions? Reach out at [nancy@kidsindanger.org](mailto:nancy@kidsindanger.org)

## Project Scope

The non-profit, Kids in Danger (KID), along with Dr. Quinlan at Rush Hospital are investigating possible causes to Sudden

Design Concept

# Validation

Validation testing was performed to prove the device could provide repeatable results and that the qualitative assessment of a mattress

Unexpected Infant Death (SUID). The team is exploring if there is a correlation between mattress firmness and SUID. Currently, the idea of Infant Safe Sleep is promoted, as seen in Figure 1, but the hope for this collaboration is for mattress manufacturers to have a new standard to follow to help solve this problem.


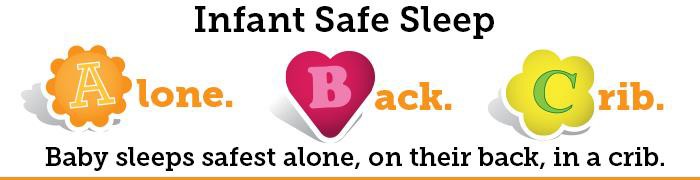


**Figure 1:** Guidelines encouraged by KID for safest sleep for infants [1]

- 3600 infants die annually in the United States from SUID

**Initial Design from Previous Semester’s 450 Team**

**Updated Slip Fit Design Final Acrylic Model**

Our concept development started with creating a morphological chart to generate new design ideas for each of the key components of the previous semester’s design. We used a Pugh Chart to select our updated slip fit design, but due to manufacturing difficulties we had to repeat the process to design the final acrylic model.

Concept Development

(soft/firm) corresponds to the quantitative measurements.

Procedure

1. Label 4 points that will be used for testing on the mattress.
2. Zero the device on a flat, rigid, surface.
3. Place the cylinder (with no weight added) at the first position (corner) and record the indentation. Take measurements immediately, 30 seconds, and 1 minute after when the indent has come to an equilibrium position.
4. After taking 3 measurements in the first position, move the device to the second position (L) and take the same 3 measurements. Then do the same at positions 3 (Center) and 4 (W).
   1. This will account for the hysteresis because there will be 3 minutes in between each measurement.
5. Repeat 3 and 4 twice more at each position with just the cylinder in order

to test the repeatability of the device.

- A quantifiable mattress rating may reveal correlation between firmness and SUID, but no such standard exists today


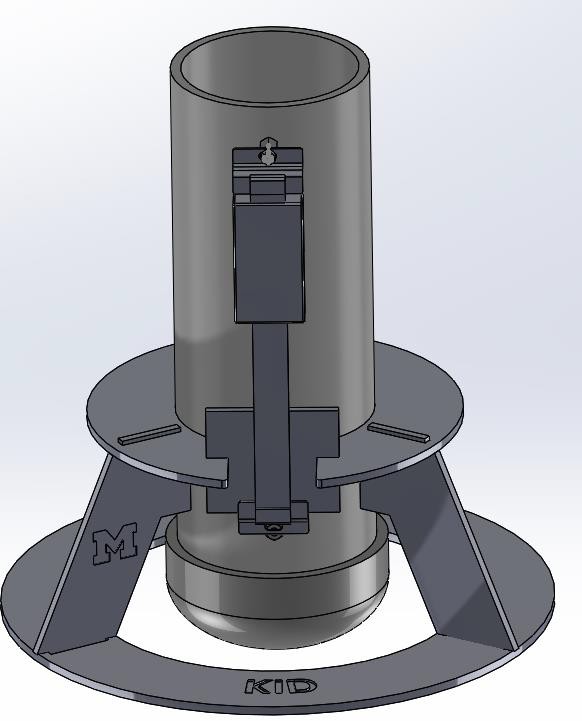

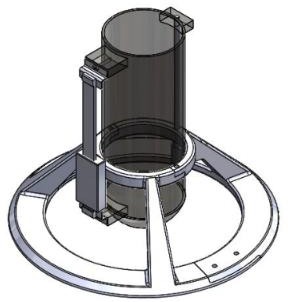

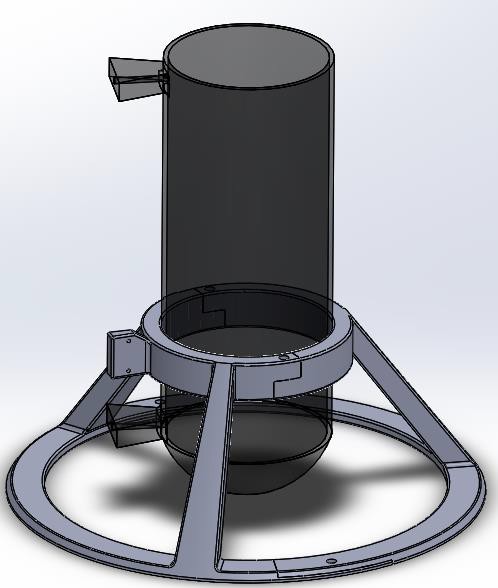


- Babies that do not have control of their neck/head yet can result in suffocation or asphyxiation due to restricted airways

## Problem Statement

The goal of this project is to create a device that measures the

Initial Design from Previous Semester’s 450 Team: This design was given to us from the team who worked on this project in W2018 semester. This design had complaints of high friction and low durability.

Updated Slip Fit Design: Contact with our sponsors and consultation of the previous team’s final report gave us some areas for improvement to make on the design. Our new slip fit version was designed to help with vertical alignment of the cylinder and a filled shell in the rings were made to increase stability. This design took over 4 days to print on a SLA 3D Printer.

Final Acrylic Model: Our final acrylic model can be laser cut in under 10 minutes. The design is cut from a flat piece of acrylic and assembled similar to the concept of puzzle pieces. We used acrylic glue to glue the pieces together and make the design more stable.

Final Design

1. Repeat 3 - 5 at each weight from ½ lb to 6.25 lbs in ¼ lb increments

Experimental Results

1.3

1.2

Mattress Displacement (in)

1.1

1

indentation of a baby’s head into a mattress to be used for investigating possible factors contributing to SUID. With this device, Child Death Scene investigators and Researchers want

- ID has a 0.05” slip fit to provide vertical alignment for the cylinder
- 3 slots in the top ring allow for the three arms to connect


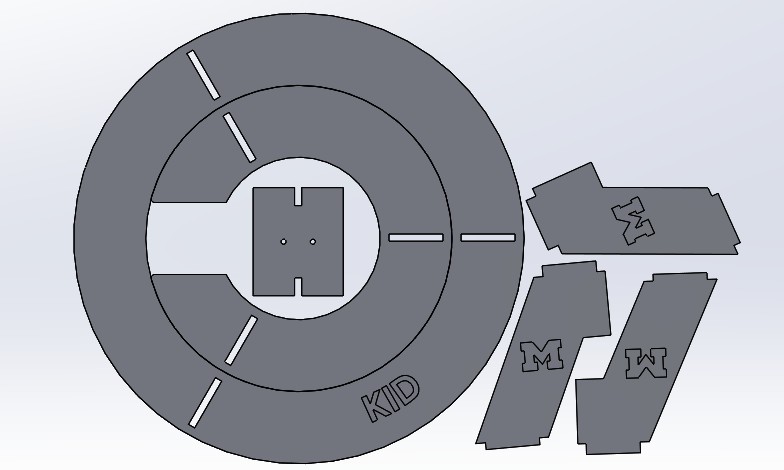


1

2

4

3

0.9

0.8

0.7

t = 0 seconds

t = 30 seconds t = 60 seconds

to quantify the softness/firmness of a child’s mattress

User Requirements and Specifications

1

Top Ring

Bottom Ring

Acrylic

- 3 slots in the bottom ring allow the 3 arms to connect
- OD = 12 inches
- Pieces that connect the top and bottom ring.

0.6

0.5

0

3

1 2 3 4 5 6

2

Weight Added (lbs)

The user requirements were used to determine engineering specifications targeting babies 2-4 months old due to lack of

head control at this age.

5

6

**Figure 3**: Laser Cut Orientation


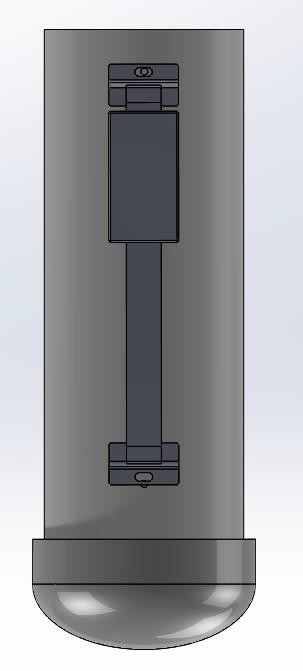

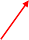


6

5

7

4


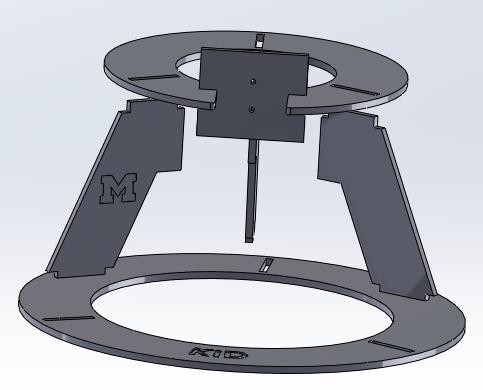


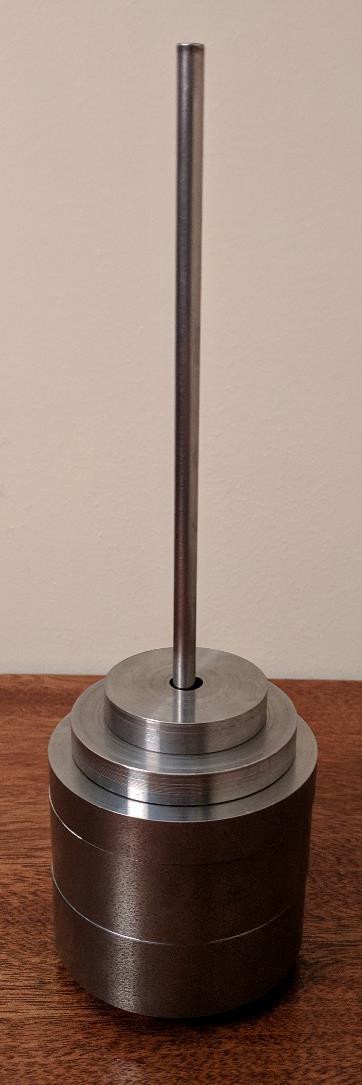


8

**Figure 4**: Exploded View

7

Arm

Device Mount

Measuring Device

Standoff PVC

Cylinder

Weight Set

- Inner flat part of arm supports cylinder with 0.05” slip fit
- Slides onto the top ring from the outside of the ring
- Holes instead of slots to ensure vertical alignment
- 0 to 4” Remote Display of Measurements [in, mm, cm]
- 0.0005” Resolution, LCD Display
- Threaded hex standoff used to attach measuring device to PVC
- 2 standoffs to control vertical position of measuring device
- 12 inches in length
- Rounded bottom cap to mimic shape of a baby’s head
- Manufactured steel weights in ¼, ½, 1 and 2 lb increments
- 6.25 ± 0.01 lb in total
- Welded 0.5 lb rod for easy add/remove of weights

**Figure 7:** Testing Results on the Spring Mattress in the Center Position

Results from our testing show that the displacement sinks to a stable depth by 30 seconds after the weight is added. We verified this by recording an additional measurement at 60 seconds which produced a value that was nearly identical to the 30 second value. Over multiple iterations of testing, the data showed that our device is more accurate at the higher weights we tested, which are closer representations for the weight of a child’s head.

| **Mattress Type** | **Position on Mattress** | **Displacement at 0.5 lbs [in]** | **Displacement at 3 lbs [in]** | **Displacement at 6 lbs [in]** |
| --- | --- | --- | --- | --- |
| Spring, 5 in thick | Center | 0.66 ± 0.08 | 0.96 ± 0.09 | 1.21 ± 0.07 |
|  | L | 0.66 ± 0.07 | 0.96 ± 0.08 | 1.23 ± 0.07 |
|  | W | 0.61 ± 0.09 | 0.94 ± 0.09 | 1.22 ± 0.08 |
|  | Corner | 0.61 ± 0.10 | 0.89 ± 0.10 | 1.31 ± 0.09 |
| Foam, 3 in thick | Center | 0.23 ± 0.04 | 0.36 ± 0.03 | 0.50 ± 0.03 |
|  | L | 0.23 ± 0.03 | 0.34 ± 0.04 | 0.55 ± 0.02 |
|  | W | 0.23 ± 0.04 | 0.33 ± 0.04 | 0.48 ± 0.03 |
|  | Corner | 0.22 ± 0.06 | 0.39 ± 0.05 | 0.54 ± 0.04 |

**Table 2:** Comparison of indentations on a firm foam mattress versus a softer composite mattress with springs

## Future Work

**Table 1:** User Requirements and Specifications for the Mattress Measurement Device with their assigned priority

| **User Requirements** | **Specification** | **Priority** |  |
| --- | --- | --- | --- |
| Resemble the size of a baby’s head | 14 – 17.5 inches in  circumference [2] | High |  |
| Resemble the weight of a baby’s head | 3 – 6 pounds of force [2] | High |  |
| Device must be portable | < 24 in. x 24 in. x 24 in.  < 25 pounds [3] | High |  |
| Take accurate measurements | Resolution of 1/16” or smaller | Medium |  |
| Must be easy to set up | Set up time < 2 minutes | Low |  |

## Citations

1. Danger, Kids In. “Safe Sleep Tips for Infant Mortality Month.” from kidsindanger.org/2017/09/safe-sleep-tips-for-infant-mortality-month/.
2. “National Center for Health Statistics.” Centers for Disease Control and Prevention from [www.cdc.gov/growthcharts/who_charts.htm#The](http://www.cdc.gov/growthcharts/who_charts.htm#The) WHO Growth Charts.
3. 16 CFR 1219.1 - Scope, compliance dates, and definitions. (n.d.). from https:/[/www.law.cornell.edu/cfr/text/16/1219.1.](http://www.law.cornell.edu/cfr/text/16/1219.1)
4. “F15.66 on Crib Mattresses”, pg. 5, from [http://dbsiprod02.astm.](http://dbsiprod02.astm/) org/BroadcastAttachments/F1566101518_Crib_Mattresses.pdf

Cylinder

8

Measuring Device

Acrylic Base PVC Cap


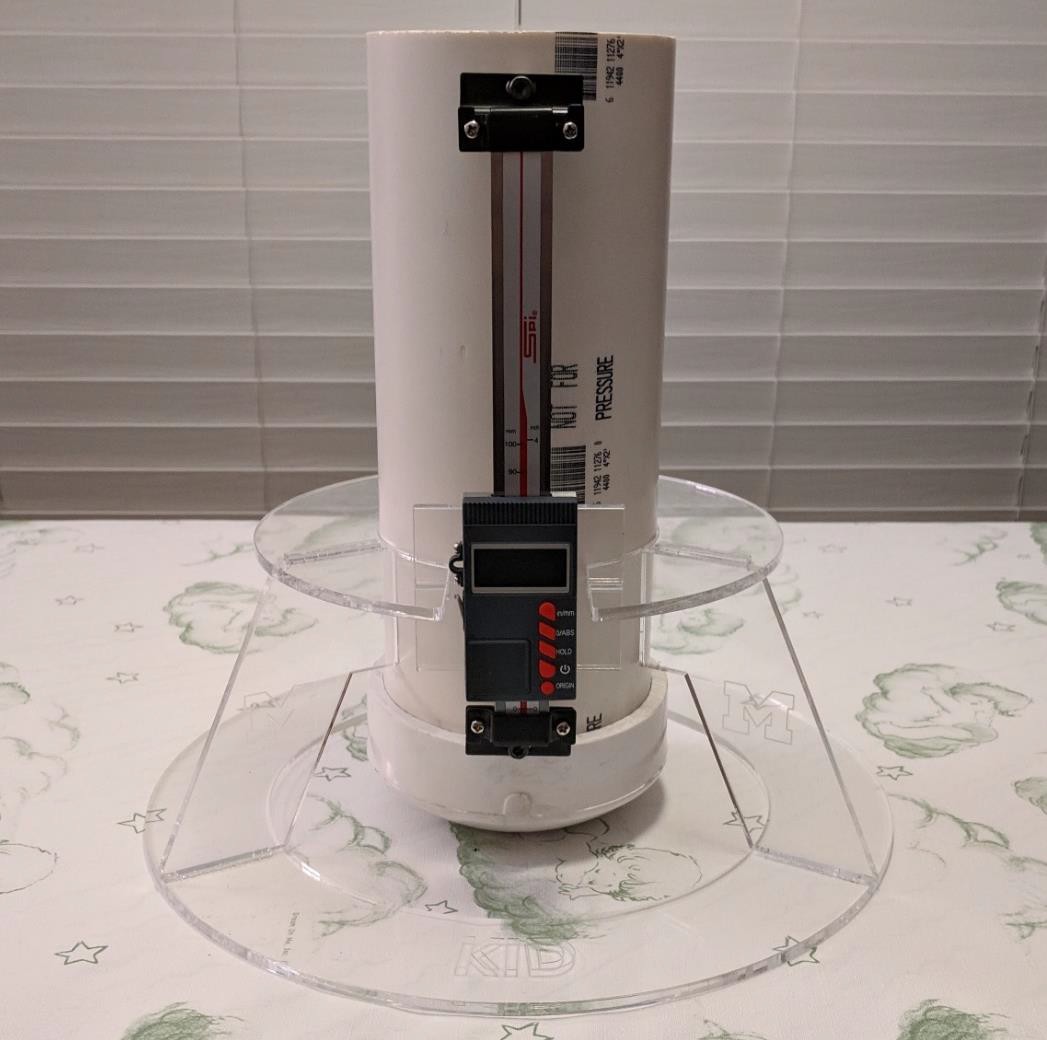


**Figure 5**: Device being tested on foam mattress in center location

To account for the broad range of mattresses available, we decided to test on both a firm mattress and a significantly softer mattress.

Testing

We compared the most commonly purchased, affordable mattresses from a variety of retailers to address the demographic where this device will be used in the future.

Two types of mattresses were selected for testing: a foam mattress (firm) and a composite spring mattress (soft).

6in

*l*

*l*/2


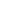

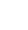

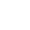

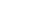


Corner

L

Center

W

**Figure 6**: Mattress Testing Placement from ASTM F15.66 on Crib Mattress Testing Standards [4]

6in

*w*/2

*w*

While our new design significantly improved the manufacturability of the device as well as the accuracy, we believe the accuracy can be further improved by:

- Decreasing the overall height of the cylinder
- Selecting a more cost-efficient and compact measurement device
- Adding a second support structure at the top of the cylinder

## Acknowledgements

This project was funded by the non-profit, Kids in Danger (KID). Special thanks to our sponsors Nancy Cowles at KID, Dr. Kyran Quinlan at Rush Hospital for their support throughout the semester. Also, thank you to Toby Donajkowski, the machine shop staff, and the entire 450 staff for their support on this project.
